# The Effects of Resveratrol in Patients with Cardiovascular Disease and Heart Failure: A Narrative Review

**DOI:** 10.3390/ijms20040904

**Published:** 2019-02-19

**Authors:** Garrison J. B. Dyck, Pema Raj, Shelley Zieroth, Jason R. B. Dyck, Justin A. Ezekowitz

**Affiliations:** 1 Canadian VIGOUR Centre, Mazankowski Alberta Heart Institute, Department of Medicine, Faculty of Medicine and Dentistry, University of Alberta, Edmonton, AB T6G 2E1, Canada; dyck@ualberta.ca; 2 St Boniface Hospital, Department of Medicine, Rady Faculty of Health Sciences, University of Manitoba, Winnipeg, MB R2H 2A6, Canada; PRaj@sbrc.ca (P.R.); SZieroth@sbgh.mb.ca (S.Z.); 3 Cardiovascular Research Centre, Department of Pediatrics, Faculty of Medicine and Dentistry, University of Alberta, Edmonton, AB T6G 2S2, Canada; jason.dyck@ualberta.ca

**Keywords:** resveratrol, CVD, heart failure

## Abstract

Cardiovascular disease (CVD) is the main cause of death globally and responsible for the second highest number of deaths in Canada. Medical advancements in the treatment of CVD have led to patients living longer with CVD but often progressing to another condition called heart failure (HF). As a result, HF has emerged in the last decade as a major medical concern. Fortunately, various “traditional” pharmacotherapies for HF exist and have shown success in reducing HF-associated mortality. However, to augment the treatment of patients with CVD and/or HF, alternative pharmacotherapies using nutraceuticals have also shown promise in the prevention and treatment of these two conditions. One of these natural compounds considered to potentially help treat HF and CVD and prevent their development is resveratrol. Herein, we review the clinical findings of resveratrol’s ability to be used as an effective treatment to potentially help treat HF and CVD. This will allow us to gain a more fulsome appreciation for the effects of resveratrol in the health outcomes of specific patient populations who have various disorders that constitute CVD.

## 1. Introduction

Cardiovascular disease (CVD) includes various disorders of the heart and/or blood vessels such as cerebrovascular disease, peripheral artery disease, coronary artery disease (CAD), deep vein thrombosis, and congenital heart disease [1]. These conditions have varying etiology, but together and among others they constitute CVD, which is the main cause of death globally [1] and responsible for the second highest number of deaths in Canada [2]. To combat the progression of these diseases and to decrease the mortality caused by them, many advancements in technology and pharmaceutical therapies have been made in recent years [3,4]. Consequently, more Canadians are now living longer with CVD but go on to develop heart failure (HF) [5]. As a result, HF has emerged in the last decade as a major medical concern. 

### 1.1. Heart Failure

In simple terms, HF can be defined as the inability of the heart to pump enough blood to the rest of the organs in the body. Of importance, the lifetime risk of developing HF for North American adults aged 45 is 20% [6] and this syndrome is expected to double over the next 1-2 decades [7,8,9,10,11,12]. In fact, it was recently reported that HF rates are increasing in Canada and the total yearly cost of HF in Canada is approximately $2.9 billion [13]. Various HF treatments exist and have shown success in reducing HF-associated mortality, such as implantable cardioverter defibrillators, heart transplant surgery, and numerous medications including ones that target the sympathetic nervous system (β-adrenergic receptor blockers), the renin-angiotensin aldosterone system (angiotensin converting enzyme inhibitors and angiotensin II receptor blockers) [14], and the pacemaker current of the sinoatrial cells (ivabradine) [15]. Furthermore, medications focusing on dual inhibition of the renin-angiotensin aldosterone system (RAAS) as well as neprilysin blockade [16] (angiotensin receptor-neprilysin inhibitor) have been shown to cause a significantly greater reduction in CV-related death, HF hospitalizations, and all-cause mortality compared to only RAAS blockade [17]. However, mortality rates following diagnosis of HF remain high [18]. Adding to this problem, most existing pharmaceutical therapies that focus on treating HF with reduced ejection fraction (HF-REF) have shown little effect in treating patients with HF with preserved ejection fraction (HF-PEF) [14]. Thus, new therapies that will help treat HF, and ultimately improve the quality of life and health outcomes of these patients is needed.

### 1.2. Nonconventional Therapies for HF

In contrast to existing pharmacotherapies that largely focus on correcting neurohumoral factors that are altered in CVD and HF [14], alternative pharmacotherapies using nutraceuticals that directly target additional factors in CVD and HF progression are being considered for the prevention and treatment of these two conditions [19,20,21]. Indeed, certain natural compounds have been shown in preclinical studies to target the underlying causes of CVD and HF such as oxidative stress [22], inflammation [23], poor endothelial function [24], and even poor left ventricle function [25]. Therefore, these nutraceuticals may potentially target aspects of CVD and HF progression missed by, or not effectively treated with, existing pharmacotherapies. In addition, these nutraceuticals are not only being considered for independent use, but as supplements to other pre-existing HF therapies as well. While early phase trials are using these natural compounds, the goal is also to provide evidence that synthetic analogs can be made from these natural compounds in order to increase the efficacy of the compound [26]. One of these natural compounds considered to potentially help treat HF and CVD and prevent their development is resveratrol. The objective of this review is to describe the evidence of the clinical utility of resveratrol on CVD and HF treatment. 

## 2. Methods

**Figure 1:**
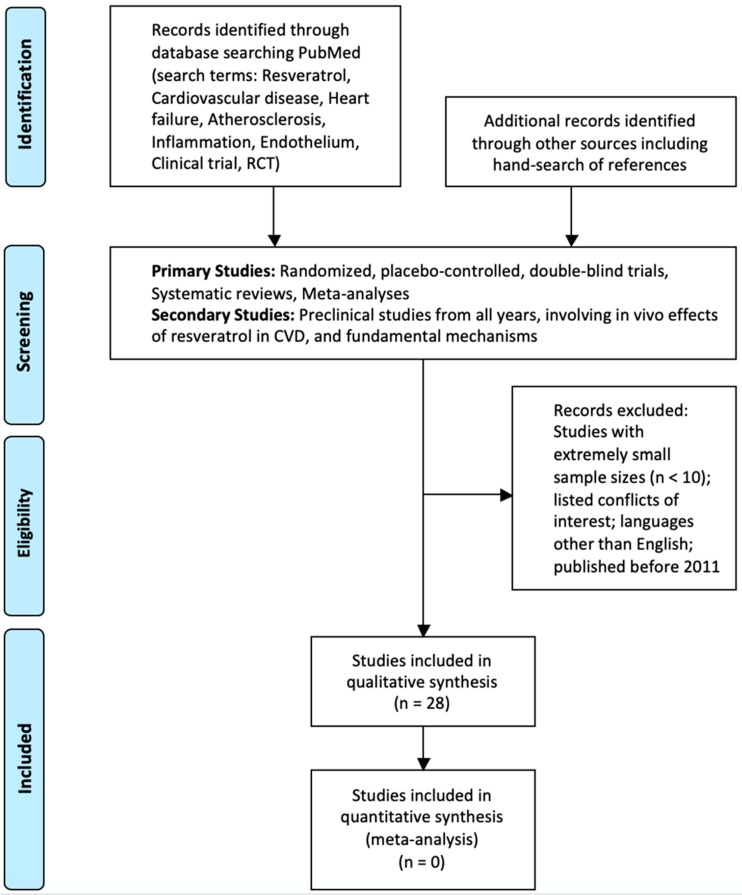
Flow Diagram Outlining the Selection of the Studies Involved in the Review.

Studies were chosen by searching PubMed for publications in English dating from 2011 to August 2018. Any randomized, placebo-controlled, double-blind trials as well as systematic reviews and meta-analyses published from 2016 through 2018 were given priority in an attempt to review the most recent literature. Searches were conducted by using keywords, often in combination, such as *clinical trial, RCT, resveratrol*, *cardiovascular disease*, *heart failure, atherosclerosis, inflammation, endothelium*. Reference lists from previous published work were also used to find related studies. The study designs were all carefully reviewed by the authors and studies with extremely small sample sizes were mostly excluded as well as studies with listed conflicts of interest. For preclinical studies and general data on disease pathogenesis, biochemistry, and pharmacology of resveratrol, older publications were used, and the searches were not limited to post-2011 (Figure 1).

## 3. Resveratrol

Resveratrol (3,5,4′-trihydroxy-*trans*-stilbene) is a polyphenolic stilbene produced by various plants when stressed [27,28]. Although individuals can obtain small amounts of resveratrol in certain foods such as peanuts, grapes, berries, etc. [29], the vast majority of the research involving resveratrol has used much higher concentrations than would occur through dietary means [30]. However, even with these higher doses, the pharmacokinetic profile of resveratrol is not optimal. For example, after oral administration of resveratrol, the absorbed resveratrol is rapidly metabolized to resveratrol metabolites that are often quickly excreted [31]. In addition, studies have shown the plasma half-life of resveratrol in humans is 4–8 h even after doses as high as 500 mg/kg are administered [32]. As a result, plasma levels are generally low, and it remains to be determined how such low concentrations of resveratrol in the blood still have important biological effects [33]. Regardless of a complete understanding of the pharmacodynamics of resveratrol, a large number of studies have demonstrated the beneficial effects of resveratrol in treating CVD in animal models [33]. While the mechanisms responsible for these effects are not clear, studies have shown resveratrol causes numerous positive effects such as decreases in inflammation [34,35], increased endothelial function [36], and a reduction in oxidative stress [37]. Most relevant to this review, resveratrol has been shown to effectively treat mice with pressure-overload-induced HF by improving diastolic function, cardiac remodeling, myocardial energetics, and vascular function, as well as reducing cardiac fibrosis [38]. However, whether or not resveratrol will have an impact on humans with HF is not clearly defined.

## 4. Clinical Evidence of the Effects of Resveratrol

Due to the preclinical effects of resveratrol, many randomized clinical trials (RCTs) have been performed over the past decade in an attempt to discover if these same benefits observed in vitro and in pre-clinical studies apply to both healthy and diseased humans. However, many of these trials use highly variable protocols and doses of resveratrol [39] and suffer from small sample sizes. Moreover, as suggested by Smolgia et al., resveratrol RCTs with healthy participants often apply paradigms only appropriate for diseased participants, which leads to potentially flawed interpretations of resveratrol as ineffective [39]. These shortcomings, as well as the variable [39] and limited [40] bioavailability of resveratrol makes it difficult to interpret if the cause of an RCT with neutral results is due to dosing and/or sample size, general lack of potency of natural resveratrol (potentially fixed through chemical techniques/analogs), or simply an ineffectiveness of the compound in treating a certain human CVD regardless of potency and dose. In addition, the variation in resveratrol RCT protocols presents a difficulty when comparing trials that attempt to test the same outcomes of resveratrol supplementation. 

### 4.1. Effects on Factors Related to the Pathogenesis of Atherosclerosis and Coronary Artery Disease

**Figure 2:**
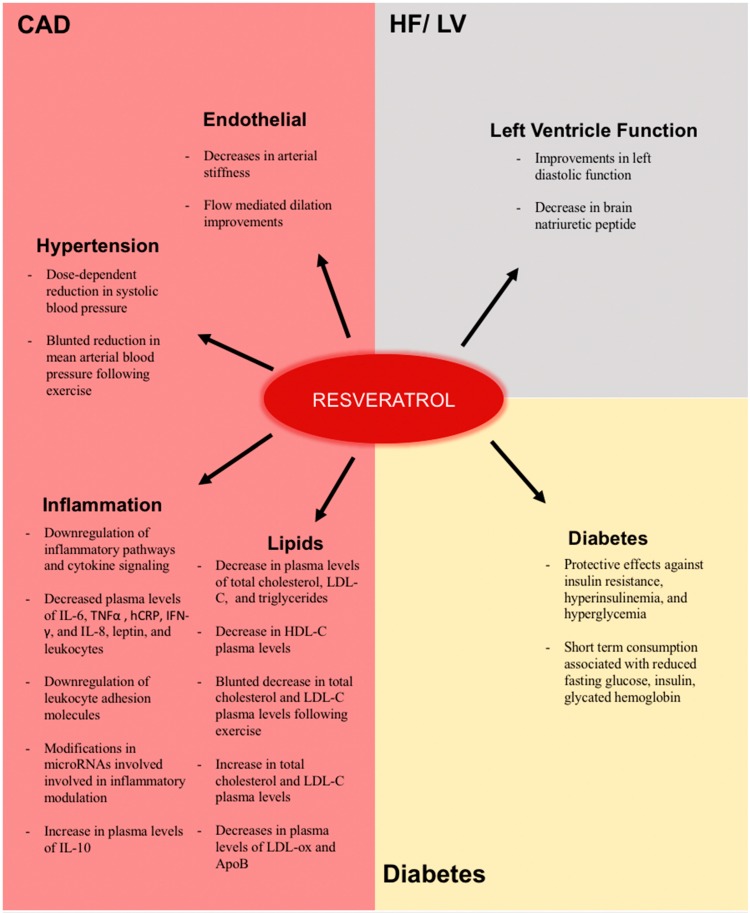
Summary of the findings from the clinical studies involving resveratrol. The key findings in the studies using resveratrol in different patient populations are summarized. The three main subgroups of disease conditions are indicated by Coronary Artery Disease (CAD), Heart Failure (HF)/Left Ventricular (LV) Function, and diabetes and the effects on different biological entities are indicated.

Atherosclerosis is often seen as a chronic low-grade inflammatory condition with a complex pathogenesis involving endothelial dysfunction, lipoprotein build up and oxidation, pro-inflammatory cytokines, and various other factors [41,42]. Atherosclerosis is also the main cause of coronary artery disease (CAD) [42], which is the most common etiology of patients with HF in developed nations [43]. Thus, to assess the efficacy of resveratrol in treating patients with HF, many clinical trials attempt to measure the effects of resveratrol on the factors related to the pathogenesis of atherosclerosis and CAD, including effects on inflammation, lipoprotein and cholesterol metabolism, and endothelial function (Table 1; Figure 2).

### 4.2. Effects in Inflammation

The link between vascular inflammation and risk of CVD, most notably hypertension and atherosclerosis, is well documented [72]. In atherosclerosis, the beginning stages of the development of an atherosclerotic lesion are characterized by endothelial cells beginning to express selective adhesion molecules such as vascular cell adhesion molecule-1 (VCAM-1) that promote attachment of leukocytes to the endothelium [41]. This activity is the most pronounced in damaged areas of the endothelium with disturbed flow and a low production of nitric oxide (NO) [73]. In addition, the smooth muscle cells (SMCs) in these damaged areas may produce proteoglycans that attach to lipoproteins, promote their oxidation, and increase the adhesion of leukocytes to the lesions of the arterial walls [74]. The chemically attracted leukocytes, including lymphocytes [75] and monocytes [76], then enter the intima and stimulate a local inflammatory response [41]. Stimulation factors also causes monocytes to develop into macrophage foam cells [77] and inflammatory cytokines released by T-cells promote the smooth muscle cells of the endothelium [78] to eventually form a thick extracellular matrix of SMCs and fibrin [79]. Given this role of inflammation in atherosclerosis, pro-inflammatory cytokines are often used as biomarkers to monitor changes in atherosclerosis risk and consequently the prognosis of HF after supplementation of a potential cardio-protective drug [41]. Common biomarkers related to the inflammatory response measured in resveratrol clinical trials include interleukin (IL)-6, tumor necrosis factor (TNF)α, c-reactive protein (CRP), Intercellular Adhesion Molecule 1 (ICAM-1), P selectin, and E selectin [41]. Other inflammatory cytokines also involved with cardiac diseases used in resveratrol RCTs to assess risk of atherosclerosis and HF include IL-8; mostly as a marker of negative effects) and IL-10 (anti-inflammatory and a marker of positive effects) [80]. 

Due to the detrimental role inflammation plays in CVDs and HF, the vast amount of evidence in cell and animal models that show the anti-inflammatory effects of resveratrol [81,82,83,84,85,86] has prompted various clinical trials testing if the compound exerts anti-inflammatory effects in humans. As described by Poulsen et al., a proposed mechanism for the potential anti-inflammatory effects of resveratrol in humans is an increased activation of the silent information regulator factor 2 related enzyme 1 (SIRT1) [87]. SIRT1 had been shown in vitro to protect against HF induced inflammation [88] and cells from mice with a SIRT1 knockout show increased pro-inflammatory cytokine levels [89]. Also, resveratrol is a known activator of SIRT1 in animals [90] and cultured cells [91,92]. Therefore, it follows that resveratrol could potentially decrease inflammation in humans by increasing the activity of SIRT1. However, multiple other potential anti-inflammatory mechanisms of resveratrol, including some that may be linked to SIRT1 activation, have been proposed such as a suppression of cytokine signaling [93], a suppression of major pro-inflammatory kinase expression [93], and an increase in levels of anti-inflammatory eicosanoid precursors [94]. However, the results of the clinical trials testing the effects of resveratrol on inflammation have been highly variable. Some clinical trials have found a decrease in plasma levels of inflammatory cytokines following resveratrol supplementation, including a decrease in the cytokines IL-6 [57,62,65,69,70], TNFα [57,60,62,69], high-sensitive CRP (hsCRP) or CRP [57,65,66], plasma interferon (IFN)-γ [58], and IL-8 [58] that could be linked to increased SIRT1 activity. Moreover, in a study performed by Timmers et al., 150 mg of resveratrol daily supplementation for 30 days resulted in modest reductions in plasma levels of leptin, leukocytes, and the previously mentioned IL-6 and TNFα in obese adults [69]. After genetic analysis, it was revealed that resveratrol downregulated inflammatory pathways and cytokine signaling [69]. Other studies have also found that resveratrol causes a downregulation of leukocyte adhesion molecules [57,58], modifications in microRNAs involved in modulating inflammation [56], and increased plasma levels of the anti-inflammatory cytokine IL-10 [57]. Given that these studies used highly variable resveratrol doses, treatment periods, and types of participants, these findings suggest that resveratrol given at various doses for varying treatment periods decreases inflammation in both healthy and diseased humans. However, numerous studies have also shown no effect of resveratrol on plasma levels of molecules involved in the inflammatory response, including IL-6 [44,52,55,58,61,68], TNFα [52,55,58,61,68,95], CRP [44,55,67,68,95], IL-8 [62], IL-1B [58], vascular cell adhesion molecules (VCAM) [68], P-selectin [68], and E-selectin [68]. It should be mentioned that studies showing lack of effect on inflammatory cytokine plasma levels also use variable participants, including healthy individuals and individuals with various diseases, and variable doses and treatment periods. Additionally, some studies have shown that despite a lack of effect on plasma levels of inflammatory cytokines, resveratrol still suppresses general inflammatory responses [58,70]. Therefore, it may be that in some of the previously mentioned studies showing a lack of anti-inflammatory effects of resveratrol, anti-inflammatory effects were simply missed due to the use of plasma cytokines as the only marker of anti-inflammation. Nevertheless, when the clinical evidence is taken together, the effects of resveratrol on inflammation seem to be highly variable and inconclusive. It is hard to know for certain why inconsistent and contradictory effects are observed on general inflammatory effects and even on the same inflammatory biomarker, but it has been proposed by Morton et al. that the variability may be due to the vast inconsistencies in doses used and differences in study populations [87]. In addition, recent studies showing the prominent inter-individual differences in resveratrol metabolism by the human microbiota may account for variability seen in the compound’s effects [96]. As the field evolves and new trials are initiated, resveratrol RCTs should focus on using a standardized range of doses so that results can be more easily compared and pre-screening the gut microbiota of participants prior to a trial may be helpful in revealing if the variations seen in resveratrol metabolism result in significant variations in its physiological effects (Table 1; Figure 2).

### 4.3. Endothelial Effects

The endothelium plays an essential and dynamic role in the cardiovascular system. The endothelial cells not only control blood flow and release NO as the primary mediator of proper vascular function but also prevent aggregation of blood cells, and control permeability of substances in the plasma [97]. The endothelium also reduces inflammation through the production of natural anticoagulants [97]. With regard to atherosclerosis, endothelial dysfunction is an essential factor in its pathogenesis [98]. Therefore, a dysfunctional endothelium is associated with high blood pressure [97], inflammation [99], CAD [98], and eventual HF [97,99]. Studies performed in vitro on human cells or animal models have shown that resveratrol has positive effects on endothelium function, including an up regulation of endothelial nitric oxide synthase (eNOS) [100], which is the primary enzyme that produces NO for the vascular system, and a decrease in the uncoupling of eNOS to reduced oxidative stress [100]. These results, as well as many others, have led to numerous resveratrol RCTs, hoping to see mimicked preclinical endothelial effects of resveratrol supplementation on human endothelial function. Compared to the previously discussed inflammatory effects, RCT results measuring changes in endothelial function, often using flow mediated dilation (FMD) as an indicator, have been less variable. Various studies where resveratrol was administered to diseased participants show an improvement in endothelial function. These improvements were observed in participants with a previous myocardial infarction [95] and participants with metabolic syndromes [44]. A decrease in arterial stiffness (measured by Cardio Ankle Vascular Index) was also observed in participants with type 2 diabetes after resveratrol supplementation [45]. Interestingly, this study also suggests that the positive effects of resveratrol on endothelial function are more pronounced for those who likely had poor endothelial function before the trial began [45]. This is supported by a study performed on obese subjects, which showed that those with worse FMDs before resveratrol supplementation saw a more significant increase in FMD than those with a more normal initial FMD [47]. This effect is further supported by a study on hypertensive participants given resveratrol, which showed that FMD improvement was higher for participants that initially had higher low-density lipoproteins (LDL) levels than in participants that had low initial LDL levels [46]. Moreover, it should be mentioned that endothelial improvement was only observed in the female participants within this study, suggesting potential sex-related differences in resveratrol effects. Due to studies showing a sex-related differences in resveratrol metabolism by the gut microbiome [101], these metabolic differences may be a potential explanation for the results reported. However, further experimentation must be performed in order to determine if this explanation is valid. Nevertheless, in reviewing the recent RCTs involving endothelial function, there is strong evidence that resveratrol supplementation does improve endothelial function. Although the mechanism(s) by which resveratrol improves endothelial function is (are) not entirely known, it has been described by Fujitaka et al. [44]— due to the fact that SIRT1 is known to activate NO synthase and increase endothelial function, resveratrol could improve endothelial function by activating SIRT1 [102]. Like previously stated, this was a proposed mechanism for anti-inflammatory effects as well [44]. However, a RCT performed by Gliemann et al. showed that resveratrol supplementation actually blunted the increase of eNOS levels following exercise and did not activate SIRT1 [48]. In addition, several other RCTs have shown a lack of effect of resveratrol on SIRT1 activity [52,55,71]. Consequently, further directions for RCTs investigating the endothelial effects of resveratrol should have a secondary objective of measuring effects on SIRT1 activity following supplementation to see if this mechanism is valid. Generally speaking, since improved endothelial changes due to resveratrol are well documented and a molecular mechanism is not entirely known, future RCTs should also focus on identifying potential mechanisms responsible for endothelial improvement (Table 1; Figure 2). 

### 4.4. Lipoprotein and Cholesterol Effects

Disorders of cholesterol and lipoprotein metabolism are a well-known risk factor for atherosclerosis and consequently, a risk factor for CAD and HF as well [103]. In regard to the pathogenesis of atherosclerosis, conditions such as hypercholesterolemia are considered to play a large role [103]. To elaborate, LDL exposed to the macrophages of an atherosclerotic lesion get oxidized, this oxidized LDL (LDL-ox) is then able to injure endothelial cells, aiding in the progression of atherosclerotic lesions [103]. In addition, lipoprotein retention within lesions, which is increased with the presence of LDL, contributes to the formation of severe plaque build-up that may cause acute thrombotic vascular events such as myocardial infarction [103]. Moreover, hypertriglyceridemia is also associated with an increased risk of CVD [104,105]. Given the benefits of statin therapy in preventing human CVD [106,107] and the aforementioned role of high plasma LDL, triglycerides, and total cholesterol in the pathogenesis of CAD and eventual HF, statin-therapy-like effects such as a lowering of LDL, triglyceride, and total cholesterol plasma levels are often used as an indicator of cardio-protective effects in RCTs evaluating the cardiovascular effects of a drug, including in trials testing the effects of resveratrol. Likewise, since elevated high density lipoprotein plasma levels relative to LDL levels are inversely related with CAD [108], higher high-density lipoproteins (HDL) levels are often used as indicators of cardio-protective effects of resveratrol as well. In RCTs investigating the effects of resveratrol supplementation on changes in lipid profile, results appear to vary. However, multiple potential mechanisms for how resveratrol could improve the lipid profile in humans have been proposed. These include a decrease in mRNA expression of hepatic 3-hydroxy-3-methyl-glutaryl-CoA (HMG-CoA) reductase [49] (an enzyme involved in cholesterol biogenesis [109]), and an activation of SIRT1 [49], which may potentially lead to reverse cholesterol transport [110] and an amelioration in lipid profile [111]. 

Despite the ability of resveratrol to mediate these potential pathways, there is more clinical evidence suggesting no direct effect of resveratrol on plasma levels of LDL, HDL, triglycerides, and total cholesterol than evidence suggesting a significant effect. For example, multiple RCTs have shown no effect of resveratrol supplementation at various doses on lipid profile [44,45,51,52,56,62,68]. Also, numerous RCTs have reported no effect on specific aspects of the lipid profile such as on LDL cholesterol (LDL-C) [59,61], HDL cholesterol (HDL-C) [61], and triglycerides [54]. Although some RCTs have shown a decrease in plasma levels of total cholesterol [59,60,61,64], LDL-C [60,95], and triglycerides [50,64,66,69] (note that in the study performed by Militaru et al. resveratrol performed worse than calcium fructoborate at improving triglyceride levels), a meta-analysis conducted by Sahebkar et al., which included many of the previously mentioned studies, found no significant effects of resveratrol on plasma levels of LDL-C, total cholesterol, and triglycerides [67]. Interestingly, this meta-analysis on resveratrol RCTs actually found the overall effect of a decrease in HDL-C plasma concentrations [67]. A more recent meta-analysis performed by Haghighhatdoost et al. found similar results, with no effect of resveratrol on plasma LDL-C levels or HDL-C levels observed [49]. In addition, total cholesterol plasma levels were only lowered in participants with a healthy body mass index (BMI) (not in overweight and obese participants) and triglyceride levels were found to increase following resveratrol supplementation [49]. However, it should be noted that when the study done by Zortea et al. [50] was removed from this meta-analysis, the triglyceride increase became insignificant [49]. Moreover, some RCTs investigating resveratrol’s effects on lipid profile have shown negative effects such as a blunted decrease in total cholesterol and LDL-C plasma levels following exercise [48], and an increase in total cholesterol and triglyceride plasma levels [65]. Keeping all evidence in mind, especially the meta-analyses, it seems that resveratrol has a lack of effect, or even a detrimental effect, on plasma lipid profile (if the indicator of lipid profile status used is plasma levels of total cholesterol, LDL-C, HDL-C, and triglycerides). 

In contrast to the aforementioned studies, a study conducted by Tomé-Carneiro et al. on participants undergoing primary prevention of CVD, showed that resveratrol supplementation resulted in a decrease in both LDL-ox and apolipoprotein B-100 (ApoB) plasma levels (it should be noted that supplement given in this trial was not pure resveratrol and contained various other minor stilbenes) [53]. This is interesting since the study also saw little effect of resveratrol on plasma levels of LDL-C, similar to the previous clinical trials that are often used as evidence of the inefficacy of resveratrol in altering risk of atherosclerosis due to lipoproteins. However, it is argued that decreases in plasma LDL-ox and ApoB are actually more valid indicators of CVD risk reduction than a decrease in LDL-C plasma levels [112]. Therefore, this study shows that despite previous studies showing no reduction in LDL-C levels nor other changes in lipid profile following resveratrol supplementation, the compound may still reduce the risk of atherosclerosis and other CVDs related to lipoproteins via a reduction in small LDL particles (LDL-P) and consequently decreased LDL-ox as well [53]. However, further research is needed to verify these results, since another RCT by Poulsen et al. found resveratrol supplementation to have no effect on lipid oxidation [52]. Overall, the evidence on the effects of resveratrol on lipids are variable and often contradictory. Thus, further research focusing on not just lipid profile, but LDL-ox and LDL-Ps is needed to fully understand if the compound can reduce the risk of CVDs associated with poor cholesterol and lipoprotein metabolism (Table 1; Figure 2).

### 4.5. Effects on Hypertension

Hypertension is not unrelated to the previously discussed pathogenesis of atherosclerosis and CAD. In fact, hypertension is a known contributor to the development of both of these CVDs [113] and effects more than 25% of the population in developed nations [114]. Furthermore, hypertension leads to damaged myocytes, left ventricular hypertrophy (LVH), and impaired coronary reserve myocardial perfusion, all conditions known to cause HF [113]. Although multiple anti-hypertensive pharmacotherapies that decrease CVD related mortality exist [113], it has been argued that these existing therapies do not always adequately protect against resistant hypertension [115] and end-organ damage. Thus, compounds like resveratrol, which show anti-hypertensive properties in multiple animal model studies [116,117,118] and that also provide potential multiple end-organ protection [119,120,121,122] are a compelling potential treatment or co-treatment for hypertension [115] and have prompted many RCTs to investigate the clinical effects of resveratrol on blood pressure (BP). The proposed mechanisms by which resveratrol could decrease BP, mostly based on preclinical experiments, include the increase in endothelial NO production [33], reductions in vascular inflammation and oxidative damage by an increased expression of SIRT1 in the endothelial cells [33], and decreased Ca^2+^ influx [33]. However, the clinical evidence of the effects of resveratrol on BP is inconclusive. Studies that show a reduction in BP usually only find a reduction in systolic blood pressure (SBP) and not diastolic blood pressure (DBP) [44,48,67,89]. However, this may not be a limitation, since it has been suggested that SBP is more of a risk factor for CVDs than DBP [33]. Moreover, as mentioned by Beshay et al., a few of the studies done on participants with metabolic disorders that show a reduction in SBP [44,48,89] might have been due to metabolic improvements and thus, a direct vasodilating effect of resveratrol cannot be confirmed. In addition, a study conducted by Theodotou et al. on hypertensive participants showed that resveratrol can be used in addition to angiotensin-converting-enzyme (ACE) inhibitors to adequately control BP without the need of another anti-hypertensive medication [123]. 

In contrast to the studies showing a resveratrol-induced SBP and DBP or just SBP reduction, other RCTs conducted on a variety of participants (including studies performed on participants with metabolic diseases) have showed no reduction in SBP [61,71,96,97,103], mean arterial BP [65,97], or peripheral BP [103]. Additionally, meta-analyses have indicated no effect of resveratrol supplementation on SBP or DBP as well [124,125]. Interestingly, three meta-analyses investigating the anti-hypertensive effects of resveratrol have all shown a dose dependent effect of the compound on SBP [67,125]. In these analyses, resveratrol doses higher than or exactly 300 mg/d [124], higher than or exactly 300 mg/d [67], and higher than or exactly 150 mg/d [125] respectively, resulted in a more pronounced SBP reduction. This is a good indication that higher doses of resveratrol should be used in future RCTs dealing with the BP effects of resveratrol. However, it should be noted that increases in BP have been seen following resveratrol supplementation, including an increase in DBP and heart rate [97] and a blunting of reductions in mean arterial BP following exercise [48]. 

To explain the lack of effect of resveratrol on BP seen in many studies, it has been suggested that the compound likely exerts more profound effects on a hypertensive population [33]. However, since that suggestion, a RCT conducted on participants with hypertension who received 300 mg/d of resveratrol has shown no effect on BP [103]. Therefore, the clinical evidence remains inconclusive and contradicting, without a clear explanation for the variability. However, since there is strong evidence showing a resveratrol dose dependent positive relationship with SBP reductions, further RCTs focusing on the effects of resveratrol on BP should use doses at least over 300 mg/d to properly evaluate this dependency and possibly confirm the reductive effect of resveratrol on SBP at high doses (Table 2; Figure 2). 

### 4.6. Effects on Diabetes

Diabetes is one of the major comorbidities associated with HF [126] and is present in as many as 40% of patients with HF [126,127,128]. Worsening diabetes is known to cause HF independent of CAD and hypertension with overall 2 to 4 times higher rate in diabetes patients compared to non-diabetic patients, according to the Framingham Heart Study [127,129]. Diabetes may precede the development of cardiac dysfunction and HF, albeit there is also evidence that suggests HF can contribute to the development of insulin resistance and diabetes with a higher rate of incidence with increasing severity of HF [130]. The concomitant presence of diabetes and HF leads to poor prognosis and worse quality of life, hospitalization, increased readmission rate and mortality in the affected population [131]. In addition, the presence of diabetes increases the risk of myocardial infarction (MI) and stroke in HF patients [132]. The abnormalities in glycemic regulation due to insulin deficiency and resistance may directly perturb cardiac function by altering the normal myocardial energetics and contribute to HF in the absence of CAD and hypertension [133]. Other underlying diabetes-induced pathophysiological factors that cumulatively affect the myocardium include advanced glycation products, lipotoxicity, impaired calcium handling, oxidative stress, mitochondrial dysfunction, and inflammation [133]. Currently, there is a lack of clear consensus in accurately defining the term diabetic cardiomyopathy and its pathophysiology [134]. However, evidence from pre-clinical and clinical characteristics point to the role of progressive diastolic dysfunction and late systolic dysfunction in the culmination of HF [134]. In conjunction with HF medical therapy, diabetes management is also increasingly recognized as an important issue to tackle when both HF and diabetes coexist [134,135,136]. The current standard of care does not differ for HF patients with and without diabetes [134]. That being said, the advent of novel compelling information on the efficacy of new diabetic drugs such as sodium-glucose cotransporter (SGLT) 2 inhibitors in HF management may offer new opportunity for improving the prognosis and outcomes in HF patients with and without diabetes [137,138]. Interestingly, resveratrol improves insulin sensitivity and glucose metabolism in rodent and non-human primates in the setting of type 1 and type 2 diabetes, metabolic syndrome, and aging [139,140,141]. Resveratrol mediated anti-diabetic effects have been attributed to the decrease in hepatic glucose production, activation of AMPK, a master regulator of metabolism, improved glucose uptake via an increase in glucose transporter, and reduction in oxidative stress [142]. In addition, resveratrol has been shown to improve cardiac structure and function in the setting of type 1 and type 2 diabetes [143,144]. Consistent with the pre-clinical studies that reported the anti-diabetic properties of resveratrol, a few clinical trials have also reported the protective effects of resveratrol against diabetic complications such as insulin resistance, hyperinsulinemia and hyperglycemia [69,142,145,146,147,148]. Moreover, a meta-analysis of 11 studies reported that short-term consumption of resveratrol reduces fasting glucose, insulin, glycated hemoglobin, and insulin resistance in diabetes patients [149] (Table 1 and Table 2; Figure 2).

### 4.7. Effects on Heart Failure and Left Ventricle Function

Since it is likely that resveratrol may have beneficial effects in numerous CVDs that can contribute to HF and/or are comorbidities of HF, it stands to reason that resveratrol may hold promise for the treatment of clinical HF. Interestingly, numerous animal models of ischemic and non-ischemic HF have shown beneficial effects of resveratrol in HF that either prolongs survival [38], improves diastolic [38] or systolic function [150], reduces negative atrial and left ventricular remodeling [38,151,152], improves hemodynamics and cardiac energetics [153] and/or improves exercise capacity [154]. However, despite these preclinical studies, it is still unknown if resveratrol can improve HF in humans. That said, in a double-blind, placebo-controlled trial involving patients with stable coronary artery disease receiving 10 mg of resveratrol/day for 3 months, resveratrol improved left ventricle diastolic function [95]. Moreover, 20 mg of resveratrol/day administered for 60 days resulted in a significant decrease in b-type natriuretic peptide (BNP) in patients with angina pectoris, suggesting improved left ventricle function [64]. Although limited, these studies suggest that resveratrol may have a direct impact on myocardial function in humans. While this does not demonstrate that resveratrol will improve myocardial performance in patients with HF, it does provide interesting data that suggests that clinical trials in the area are warranted.

Due to the interest in the area, clinical trials have been initiated involving patients with HF. The REV-HF (Evaluating the Clinical Efficacy of REsVeratrol in Improving Metabolic and Skeletal Muscle Function in Patients with Heart Failure clinicaltrials.gov NCT03525379) is a randomized, double-blind, placebo-controlled trial evaluating the change in skeletal muscle function VO_2_ after 8 weeks of therapy in patients with HF. In addition, the RES-HF trial (RESveratrol: a Potential Anti-remodeling Agent in Heart Failure, clinicaltrials.gov NCT01914081) is a randomized, double blinded, placebo-controlled study designed to assess the clinical efficacy and safety of one year of resveratrol therapy in ischemic and non-ischemic HF patients. The primary and secondary objectives include changes in echocardiographic and patient reported outcome measures (Minnesota Living with Heart Failure Questionnaire), endothelial function, body fat and lean muscle mass as well as biomarkers of inflammation, antioxidant and NO activity. In addition, RES-HF will also help determine the safety and tolerability of high-dose long-term resveratrol treatment in HF patients and provide compelling new information on the therapeutic potential of resveratrol as an adjunct to current HF medical therapy (Table 3; Figure 2). 

## 5. Conclusions

Resveratrol presents a therapeutic agent with a novel mechanism of action that appears to benefit a variety of conditions related to CVD and HF. Ongoing studies will test the hypothesis that the addition of resveratrol in meaningful doses can help patients with CVD and/or HF.

## Figures and Tables

**Table 1:** Summary of Studies Involved in Coronary Artery Disease.

Study Done by	Study Design	Subjects	Dose and Treatment Period	Area of Interest	Primary or Key Exploratory Outcomes	Secondary Outcomes
**Atherosclerosis and Coronary Artery Disease**						
***Endothelial Function***						
Fujitaka et al., 2011 [44]	Randomized	34 patients with metabolic syndromes	100 mg of resveratrol (Longvinex; contains reseveratrol but also vitamin D3, quercetin, and rice bran phytate) daily for 3–6 months	Effects of resveratrol on the endothelial function of metabolically unhealthy patients	Increase in flow mediated dilation (FMD; i.e., endothelial function improvements).	No effect on body composition, lipid profile, interleukin-6 (IL-6) and high-sensitive C-reactive protein (hsCRP).
Imamura et al., 2017 [45]	Double blind, randomized, placebo-controlled	50 adults with type 2 diabetes mellitus	100 mg of resveratrol (BHN Corporation (Tokyo) as resveratrol-ε) daily for 12 weeks	Effects of resveratrol on arterial stiffness	Decrease in arterial stiffness (measured by decrease in cardio-ankle vascular index; CAVI).	No effects on fasting plasma glucose, HbA1c, total cholesterol, triglycerides, high-density lipoproteins (HDL) cholesterol (HDL-C) and low-density lipoproteins (LDL) cholesterol (LDL-C). Improved systolic blood pressure but no effect on diastolic blood pressure.
Marques et al., 2018 [46]	Double blind, cross-over, randomized, placebo-controlled	24 hypertensive adults	300 mg of resveratrol (Bioderm Pharmacy (Rio de Janeiro, Brazil) once daily	Cardiovascular effects of acute resveratrol dose	Improved endothelial function (FMD improvements); no effect on peripheral blood pressure (BP), Augmentation Index, and aortic systolic BP (SBP).	
Wong et al., 2013 [47]	Randomized, placebo-controlled, double-blind crossover	28 obese, otherwise healthy, adults	75 mg daily of resveratrol for 6 weeks	Effects of resveratrol on endothelial functioning of obese patients	Increase in endothelial function (FMD improvements). No effect on BP or arterial compliance.	
***Lipoprotein and Cholesterol***						
Gilemann et al., 2013 [48]	Randomized double-blind placebo-controlled	27 physically inactive aged (mean age = 65 ± 1 year) men	250 mg of resveratrol (Fluxome Inc., Stenlose, Denmark) daily for 8 weeks	Effect of resveratrol with exercise on cardiovascular health	Blunted decreases in total cholesterol, and ratio of total cholesterol/HDL levels following exercise. Blunted the phosphorylation of endothelial nitric oxide (NO) synthase (eNOS) following exercise. Blunted increase of eNOS following exercise. Decreased maximum oxygen uptake after exercise.	Blunted mean arterial pressure decreases following exercise. No effect on blood glucose, body mass index (BMI), protein expression in skeletal muscle, including for silent information regulator factor 2 related enzyme 1 (SIRT1). Blunted effects on increases in prostacyclin (PGI2) after exercise.
Haghighatdoost et al., 2018 [49]	Systematic review and meta-analysis	763 adults included in total cholesterol analysis, 728 adults included in LDL-C analysis, 777 adults included in HDL-C analysis, and 921 in serum triglyceride analysis.	Resveratrol doses ranged from 10 mg/day to 1500 mg/day with treatment periods ranging from 4 to 24 weeks	Effects of resveratrol on lipid profile	Decreased total cholesterol in subjects with normal BMI, but not those overweight or obese; No effect on LDL-C or HDL-C.	Increase in plasma triglyceride levels, this effect became insignificant when one study (Zortea et al. [50]) was removed from the meta-analysis).
Heebøll et al., 2016 [51]	Double blind, randomized, placebo-controlled	28 adults with non-alcoholic fatty liver disease	1500 mg of resveratrol (Evolva SA (Basel, Switzerland) daily for 6 months	Effects of resveratrol on symptoms associated with non-alcoholic fatty liver disease	No changes in plasma glucose, insulin, lipid profile or homeostatic model assessment (HOMA) index.	No effect of BMI, weight, waist-hip ratio, SIRT1 or AMP-activated protein kinase (AMPK) activity.
Poulsen et al., 2013 [52]	Randomized, placebo-controlled, double blinded	24 obese, otherwise healthy, males	500 mg of resveratrol (Fluxome Inc., Stenlose, Denmark) daily for 4 weeks	Effects of high dose of resveratrol	No effects on lipid oxidation, adiponectin or insulin, body composition.	No effect on BP, lipid profile, liver function, SIRT1, AMPK pathways or inflammatory biomarkers.
Tomé-Carneiro J et al., 2012 [53]	Triple-blind, randomized, placebo-controlled	75 adult patients given primary prevention of CVD	370 mg capsule with 350 mg Stilvid® (23 mg resveratrol/gram and other minor grape stilbenes) daily and 20 mg magnesium stearate and SiO_2_ (inactive) for 6 months	Cardiovascular effects of resveratrol	Decrease in apolipoprotein B-100 (ApoB) and oxidized LDL (LDL-ox) plasma levels, cannot be ruled out if resveratrol had a synergistic effect with other grape polyphenols in the capsule.	
Zare Javid et al., 2017 [54]	Randomized double-blind, placebo-controlled	43 adults with type 2 diabetes	480 mg of resveratrol [(ingredients: Polygonum cuspidatum extract (72%) with at least 60% trans-resveratrol, gelatin, microcrystalline cellulose (filler), and magnesium stearate) from Herbafit] for 4 weeks	Metabolic effects of resveratrol	Increased insulin resistance. No effect on plasma levels of fasting glucose or triglycerides.	
Zortea et al., 2016 [50]	Randomized double-blind, placebo-controlled	19 adult men with schizophrenia	200 mg of resveratrol (*trans*-resveratrol, 98% purified) daily for 30 days	Cardiovascular effects of resveratrol	Decrease in triglyceride plasma levels. No effects on serum glucose or body weight, BMI, and waist circumference.	
***Inflammatory Effects***						
Olesen et al., 2014 [55]	Randomized, double-blinded, placebo-controlled	43 healthy, physically inactive, elderly, men	250 mg of resveratrol (Fluxome Inc., Stenlose, Denmark) daily with and without exercise for 8 weeks	Effects of resveratrol on skeletal muscle inflammation both alone and with exercise	No anti-inflammatory effect without exercise, including no plasma level changes of c-reactive protein (CRP), IL-6, or tumor necrosis factor (TNF)α. A blunting on anti-inflammatory effect with exercise training.	No endurance effects, effects on SIRTI or AMPK pathways but an overall decrease in acetylation level. No effect on protein content of skeletal muscle, or protein carbonylation.
Tomé-Carneiro J et al., 2013 [56]	Triple-blind, randomized, placebo-controlled	75 stable CAD patients	370 mg capsule with 350 mg Stilvid® (23 mg resveratrol/gram) daily and 20 mg magnesium stearate and SiO_2_ (inactive) for 1 year	Cardiovascular effects of resveratrol	Increase in serum adiponectin levels. Decrease in plasminogen activator inhibitor-1 (PAI-1) plasma levels. General suppression of peripheral blood mononuclear cell (PBMC) -mediated inflammatory pathway, however no changes in levels of TNFα, IL-6, or IL-10.	
Tomé-Carneiro J et al., 2012 [57]	Triple-blind, randomized, placebo-controlled	75 adults undergoing primary prevention for CVD	8 mg of resveratrol daily for 1 year	Inflammatory effects of resveratrol	Decrease in hsCRP, TNFα, plasminogen activator inhibitor type 1, or IL-6/IL-10 ratio. Increase in IL-10 and adiponectin plasma levels. Decrease in soluble intercellular adhesion molecule plasma levels.	
***Various Measures Relating to Atherosclerosis***						
Agarwal et al., 2013 [58]	Double blind, randomized, placebo-controlled	41 healthy adult subjects	400mg trans-resveratrol (98% pure, sourced from *Polygonum Cuspidatum)*, 400mg grape-skin extract, and 100mg quercetin daily for 4 weeks	Effects of resveratrol on endothelial function and atherosclerosis	Reduction in mRNA expression of vascular cell adhesion molecule (VCAM), intercellular adhesion molecule 1 (ICAM-1) and IL-8. Reduction in plasma interferon (IFN)-γ. No effect on IL-1β, IL-6, and TNFα plasma levels, however overall endothelial cell cytokine activation decreased.	Reduction in fasting insulin concentrations.
Bhatt et al., 2012 [59]	Open-label, randomized, controlled	57 male adults with type 2 diabetes mellitus	250 mg of resveratrol (Biofort; Biotivia Bioceuticals International, New York, NY, USA) daily for 3 months	Cardiovascular and metabolic effects of resveratrol	Decreases in hemoglobin A1c (HbA1c), SBP, total cholesterol, and total protein.	No significant change in LDL plasma levels or body weight.
Chen et al., 2015 [60]	Double blind, randomized, placebo-controlled	60 adults with non-alcoholic fatty liver disease	300 mg of resveratrol (brand not provided) for 3 months	Metabolic effects of resveratrol	Decreased LDL-C and total cholesterol, glucose, or inflammatory cytokines. Improved insulin resistance. Increased adiponectin levels.	
Huang et al., 2016 [61]	Systematic review and meta-analysis	681 adults	Resveratrol doses ranging from 8 mg/day to 3000 mg/day With a duration of treatment ranging from 2 weeks to 6 months	Effects of resveratrol on cardiovascular disease risk factors in overweight and obese adults	Decreases in blood plasma total cholesterol levels (no change in LDL-C and HDL-C levels were observed). Decreases in SBP and no effect on DBP. No effect on fasting glucose levels, except when stratified for patients with metabolic syndrome. No effect in inflammatory biomarkers IL-6 and TNFα plasma levels.	No effect on body weight. In higher resveratrol doses (more than or exactly 300 mg per day) significant decreases in SBP, fasting insulin, fasting glucose, and total cholesterol was seen. In lower doses (less than 300 mg daily) reductions in HbA1c were observed. Decreases in total cholesterol, glucose, and HbA1c were more significant for participants who took resveratrol for more or equal to 3 months. Decreases in fasting insulin plasma levels were more significant for patients who took resveratrol for less than 3 months.
Macedo et al., 2015 [62]	Double-blind, placebo-controlled study	60 healthy adults	100 mg of resveratrol (*Polygonum cuspidatum* provided by Farmel Pharmacy (São Paulo, SP, Brazil)) daily for 3 months with routine fitness tests	Effects of resveratrol of participants undergoing a fitness test	No effect on total lipid profile. Reduction in IL-6 and TNFα plasma levels. No effect on IL-8 plasma levels. No antioxidant effects observed.	
Mendez-del Villar et al., 2012 [63]	Double blind, randomized, placebo-controlled	24 adults with metabolic syndromes	1500 mg of resveratrol daily for 90 days	Cardiovascular and metabolic effects of resveratrol	Decreases in total weight, BMI, fat mass, and waist circumference. Decreases in total insulin secretion and area under the curve (AUC) of insulin.	
Millatru et al., 2013 [64]	Randomized, double-blinded, active-controlled, parallel	87 adults with stable angina pectoris	20 mg of resveratrol daily or 20 mg of resveratrol daily and 112 mg of calcium fructoborate (CF) daily (shown to slow down the breakdown of resveratrol in the digestive system)	Cardiovascular effects of resveratrol alone and in combination with CF	In combination with CF, decreased N-terminal pro b-type natriuretic peptide (NT-proBNP) plasma levels. Decreased plasma levels of total cholesterol and triglycerides. Decreased number of angina episodes.	Less effective than CF alone in decreasing LDL plasma levels and increasing HDL plasma levels.
S. Bo et al., 2016 [65]	Double blind, randomized, placebo-controlled	179 adults with type 2 diabetes	Either 500 mg or 40 mg of resveratrol (provided by Biotivia Bioceuticals (International SrL, Italy) daily for 6 months	Cardiovascular effects of resveratrol	No changes in CRP levels.	Slight increase in plasma levels of total cholesterol and triglycerides. No changes in BMI, waist circumference, arterial blood pressure, IL-6, fasting glucose, HbA1c, and insulin.
S. Bo et al., 2013 [66]	Double blind, randomized, placebo-controlled	49 healthy adult smokers	500 mg of resveratrol (provided by Biotivia Bioceuticals (International SrL, Italy)) daily for 30 days	Anti-inflammatory and antioxidant effects of resveratrol	Reduction in CRP plasma levels.	Reduction in triglyceride plasma levels. Increase in Total Antioxidant Status.
Sahebkar et al., 2013 [67]	Systematic review Meta-analysis	600 adults	Resveratrol doses ranged from 8 mg/day to 1500 mg/day. Treatment periods ranged from 60 days to one year.	Effects of resveratrol on CRP plasma levels and other cardiovascular risk factors	No effect on total cholesterol plasma levels. No effect on plasma triglyceride or glucose concentrations. Slightly reduced HDL-C plasma concentrations.	No effect on CRP plasma levels. No effect on BP.
Van der Made et al., 2015 [68]	Double blind, randomized, placebo-controlled, cross over	45 overweight or slightly obese adults	150 mg of resveratrol (resVida) daily for 4 weeks, followed by 4 weeks wash out, and another 4 weeks of supplementation	Cardiovascular and metabolic effects of resveratrol	No differences in serum apolipoprotein A-I (apoA-I) or apoB-100 concentrations.	No effect on the levels of metabolic risk factors in plasma (including LDL and HDL). Increase in diastolic BP and heart rate. No effect on mean arterial pressure, SBP, or insulin concentrations. No effect on biomarkers of inflammation (hsCRP, IL-6, E-selectin, thromobomodulin, P-selectin or TNFα). No effect on ICAM-3, soluble ICAM-1 (sICAM-1), soluble vascular cell adhesion molecule-1 (sVCAM-1) plasma levels.
Timmers et al., 2011 [69]	Randomized double-blind crossover design	11 obese, but otherwise healthy, patients	150 mg of 99% pure trans-resveratrol (resVida™) daily for 30 days	Effects of resveratrol on metabolism	Decrease in alanine transaminase plasma levels. Lower leptin and leukocyte plasma levels. Decrease in IL-6 and TNFα plasma levels. Lower HOMA index. Lower plasma levels of triglycerides. No changes in plasma non-esterified fatty acids. Higher respiratory quotient. Lower mean arterial pressure and SBP and no effect on DBP. Lower non- esterified fatty acids and free glycerol in the late postprandial phase, however no effect on postprandial triglycerides and lactate response. No difference on ethanol in/out ratios or blood flow in adipose tissue and skeletal muscle. No effect on interstitial glucose, pyruvate and lactate responses in adipose tissue. No effect on interstitial glucose, pyruvate, lactate and glycerol concentrations in skeletal muscle. In the postprandial phase energy expenditure was lower and fat oxidation decreased. Upregulation of mitochondrial oxidative phosphorylation in vastus lateralis muscle cells. Down regulation of cytokine signaling in vastus lateralis muscle cells. Increased phosphorylated AMPK in muscle cells. No effect on mitochondrial DNA copy number. No effect on mitochondrial density. Overall mitochondrial activity increased. No effect on mitochondrial recovery following moderate exercise. Lower storage of lipids within the liver and higher storage in type 1 muscle fibres.	
Tomé-Carneiro J et al., 2013 [70]	Triple-blind, randomized, placebo-controlled	35 adult males with type 2 diabetes or hypertension	370 mg capsule with 350 mg Stilvid® (23 mg resveratrol/gram and other minor grape stillbenes) daily and 20 mg magnesium stearate and SiO_2_ (inactive) for 1 year	Cardiovascular effects of resveratrol	A downregulation of inflammatory cytokines. No effect on SBP, DBP, weight, lipid profile, glucose plasma levels, HbA1C, hsCRP, adiponectin, PAI-1, TNFα, and IL-10. Decrease in IL-6 plasma levels. Modifies microRNA (miR)s involved in inflammatory modulation.	
Yoshino et al., 2012 [71]	Randomized, double-blind, placebo-controlled	29 non-obese, normal glucose tolerant, woman	75 mg of resveratrol 99% pure trans-resveratrol [(resVida™ from DSM Nutritional Products, Ltd.)] a day for 12 weeks	Effects of resveratrol on metabolically healthy individuals	No effect on body composition, insulin sensitivity, AMPK or SIRT1 pathways.	

**Table 2:** Summary of Studies Involving Hypertension.

Study Done by	Study Design	Subjects	Dose and Treatment Period	Area of Interest	Primary or Key Exploratory Outcomes	Secondary Outcomes
**Hypertension**						
Fogacci et al., 2018 [124]	Meta-analysis	681 adults	Several doses for a time period ranging from 30 days to six months	Effects of resveratrol on SBP and DBP and mean arterial pressure	No significant effect on SBP and DBP or mean arterial pressure.	Lower DBP in higher doses (more or exactly 300 mg/day) and with diabetic patients.
Liu et al., 2015 [125]	Meta-analysis	274 adults	Doses ranging from 16 mg daily to 1000 mg daily with supplementation periods ranging from 30 days to 12 months	Effect of resveratrol on SBP and DBP	No significant reduction of SBP or DBP.	Resveratrol was more effective at reducing SBP in higher doses ( ≥150 mg daily).
Theodotou et al., 2016 [123]	Double blind, randomized, placebo-controlled	97 patients with hypertension	50 mg of resveratrol (Elevlor) daily for six months	97 patients with hypertension	Resveratrol supplementation with Dapril reduces BP to normal levels.	Resveratrol prevents liver damage.

**Table 3:** Summary of Studies Involving Heart Failure and Left Ventricular Dysfunction.

Study Done by	Study Design	Subjects	Dose and Treatment Period	Area of Interest	Primary or Key Exploratory Outcomes	Secondary Outcomes
**Heart Failure/LV Function**						
Maygar et al., 2012 [95]	Double blind, randomized, placebo-controlled	40 adults who had a previous myocardial infarction	10 mg of resveratrol daily for 3 months	Cardio-protective effects of resveratrol	Improvement in left ventricular diastolic function, endothelial functioning (FMD improvements). Decrease in plasma LDL levels. No effect on HbA1c, TNF-alpha, or CRP.	
